# In-Depth Genomic Analysis: The New Challenge in Congenital Heart Disease

**DOI:** 10.3390/ijms25031734

**Published:** 2024-02-01

**Authors:** Francesco Nappi

**Affiliations:** Department of Cardiac Surgery, Centre Cardiologique du Nord, 93200 Saint-Denis, France; francesconappi2@gmail.com or f.nappi@ccn.fr; Tel.: +33-149334104; Fax: +33-149334119

**Keywords:** congenital heart disease, first heart field, second heart field, whole-exome sequencing, whole-genome sequencing, loss-of-function variant, copy number variants, gene variants, deletion, de novo mutations

## Abstract

The use of next-generation sequencing has provided new insights into the causes and mechanisms of congenital heart disease (CHD). Examinations of the whole exome sequence have detected detrimental gene variations modifying single or contiguous nucleotides, which are characterised as pathogenic based on statistical assessments of families and correlations with congenital heart disease, elevated expression during heart development, and reductions in harmful protein-coding mutations in the general population. Patients with CHD and extracardiac abnormalities are enriched for gene classes meeting these criteria, supporting a common set of pathways in the organogenesis of CHDs. Single-cell transcriptomics data have revealed the expression of genes associated with CHD in specific cell types, and emerging evidence suggests that genetic mutations disrupt multicellular genes essential for cardiogenesis. Metrics and units are being tracked in whole-genome sequencing studies.

## 1. Current Knowledge

**Figure 1:**
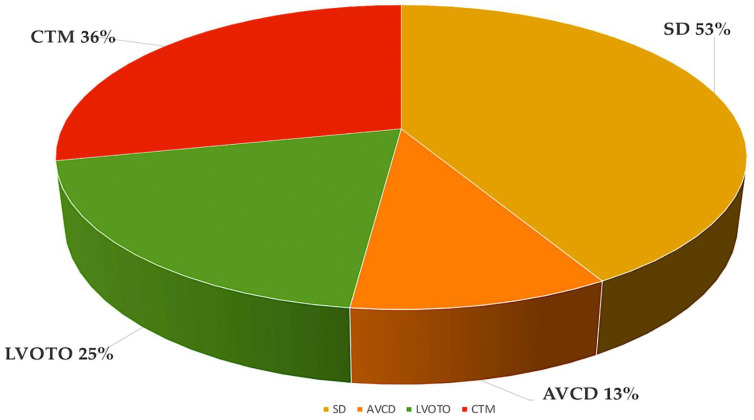
The most frequent forms of CHD from the Paediatric Cardiac Genomics Consortium, with percentages indicating the prevalence of the malformation in the patient population. It is important to note that the percentages exceed 100% as a result of concomitant structural cardiac abnormalities in the individuals Abbreviations: AVCD, atrioventricular canal defect; CHD, congenital heart disease; CTM, conotruncal malformation; LVOTO, left ventricular outflow tract obstruction; SD, septal defect. Refs. [17,18].

**Figure 2:**
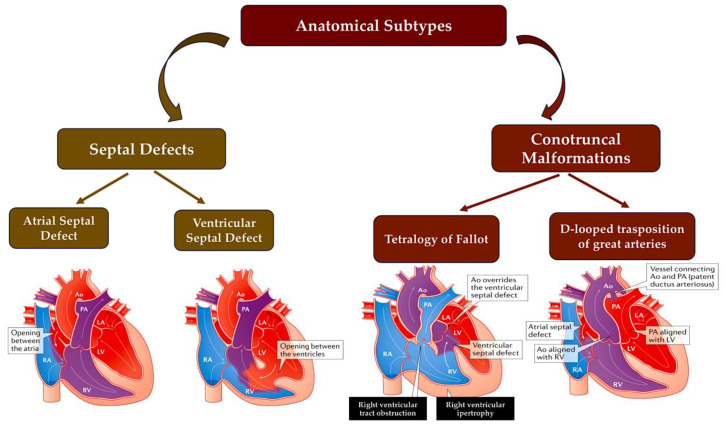
A simplified scheme of CHD defects divided according to anatomical subtype and their corresponding oxygen saturation levels. Anomalies in the communication between the left and right ventricles, such as ASD or VSD or atrioventricular canal defects, usually result in acyanotic states due to a left-to-right shunting of oxygen-rich blood into the pulmonary circulation. LVOTOs can range from isolated aortic stenosis to a combination of anomalies, such as hypoplastic left heart syndrome with mitral and aortic stenosis, which can cause cyanosis. Conotruncal malformations, such as TOF and TGV, are often associated with cyanosis due to the shunting of deoxygenated blood into the systemic circulation. This information is from the Paediatric Cardiac Genomics Consortium. Abbreviations: Ao, aorta; LA, left atrium; LV, left ventricle; PA, pulmonary artery; RA, right atrium; RV, right ventricle; TGV, transposition of great vessel; TOF, tetralogy of Fallot. Refs. [17,18].

Congenital heart disease is widely acknowledged as the most frequent and frequently serious abnormality present at birth, with a prevalence of 6–13 per 1000 newborn infants [1,2,3,4,5,6]. CHD encompasses a broad spectrum of heart abnormalities, ranging from a single defect, such as an atrial septal defect (ASD) or a ventricular septal defect (VSD), or an isolated dysplastic valve, to more complex disorders. Cardiac conditions such as tetralogy of Fallot (TOF) or hypoplastic left heart syndrome (HLHS) are characterised by multiple defects. Figure 1 and Figure 2 show the improved operative and management techniques that have led to a significant reduction in the mortality rate of CHD. These techniques are used to treat critical malformations that require early intervention for survival. Over 90% of CHD patients survive into adulthood [7], resulting in a higher prevalence of CHD in the general population. In addition, the extended lifespan of patients with CHD has prompted greater acknowledgement of the related comorbidities. Childhood extracardiac structural or functional abnormalities and neurodevelopmental delays occur in approximately 13% of newborns with CHD [3,8]. CHD is considered syndromic if other diagnoses are likely to have resulted from the same cause. Syndromic CHD often results in complex heart malformations that can cause lifelong health problems affecting various bodily systems.

Therefore, an understanding of the causes and underlying pathways of CHD can provide valuable information about both the healthy and abnormal development of multiple organs.

### Genetics

Due to significant technical advances in human genome research, genetics has now been found to play a crucial role in the development of CHD. Evidence initially emerged from examining patients with syndromic CHD through karyotyping, which identified aneuploidies such as trisomy 13, 18, or 21 (Down syndrome) and monosomy X (Turner syndrome) in approximately 12% of CHD patients [9]. Cytogenetic analyses and genomic arrays have aided in the understanding of subchromosomal structural rearrangements. These analyses have highlighted frequent 3 Mb deletions, which are also associated with DiGeorge (velocardiofacial) syndrome [10,11,12,13]. Deletions of 22q11.2, as determined by fluorescence in situ hybridisation (FISH) [14,15] and targeted amplification [16], are present in approximately 2% of all cases of congenital heart disease and in 13% of individuals with selected heart malformations. About 25% of sporadic CHD cases are caused by the coexistence of karyotype or microarray-detected abnormalities [17,18]. The preliminary detection of the monogenic causes of familial CHD has been facilitated by genome-wide linkage causes of familial CHD, encompassing variations in genes encoding transcription factors and transcriptional regulators of genes involved in heart growth [19,20]. Enhanced comprehension of the human genome at the base pair level, along with advancements in sequencing technologies, has facilitated the identification of genes linked to CHD. Harmful variants, such as missense mutations, loss-of-function mutations (LOFs), small insertions or deletions, copy number variants (CNVs), and structural defects, can be identified using advanced whole-exome sequencing (WES) and whole-genome sequencing (WGS) platforms, along with bioinformatic tools and large sequencing datasets from population-based studies. These techniques have been used to identify harmful variants present in individuals with a family history of congenital heart disease, new mutations in CHD subjects with healthy parents, and a significantly higher frequency of mutations in approximately 20% of CHD patients in large-cohort studies [18,19,20,21,22,23,24,25].

**Figure 3:**
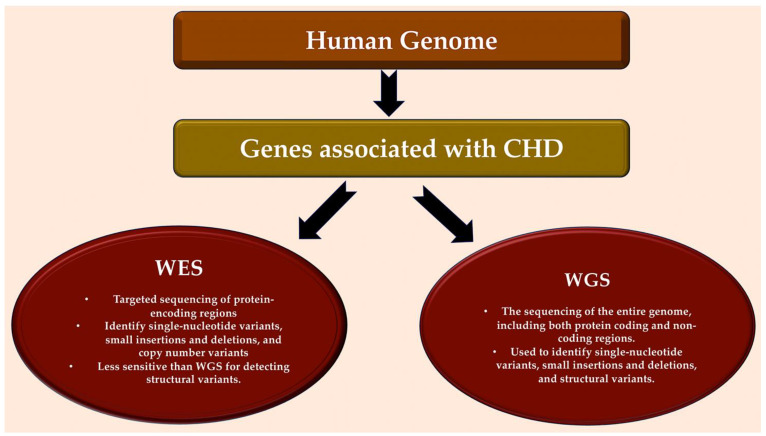
Detection of genes associated with CHD in human genome. Bioinformatic analytical tools such as WES and WGS platforms can be used to identify deleterious missense and LOF variants, small insertions or deletions, CNVs, and structural variants in large, aggregated, population-based sequencing datasets. Abbreviations: CHD, congenital heart disease; CNV, copy number variant; LOF, loss-of-function; WES, whole-exome sequencing; WGS, whole-genome sequencing. Refs. [18,19,20,21,22,23,24,25].

In 45% of patients with CHD, these methods can detect harmful coding mutations in genes that are definitively or potentially linked to CHD [18,19,20,21,22,23,24,25]. Genes that are strongly associated with CHD exhibit mutations that significantly co-segregate within families affected by CHD, are significantly more prevalent in unrelated patients with CHD compared to control groups, or cause heart defects in individuals with CHD linked to syndromes. CHD candidate genes share common features, but their properties lack statistical significance for definitive categorisation. Different types of variants, such as aneuploidies, copy number variants, structural genetic variations, and LOF variations, affect the dosage of genes related to CHD. However, deleterious missense variants can preserve the physiologic gene dosage whilst impairing the function of the encoded protein [21,24]. Please refer to Figure 3.

Genes linked to CHD provide new knowledge about the essential regulatory molecules and pathways involved in cardiogenesis, complementing and extending the wealth of information gained from studying heart development in experimental models. Studying spontaneous gene variants in sporadic CHD can reveal novel genes implicated in cardiogenesis while providing detailed information [17,21,24]. Clinical evaluations enable the identification of a wider range of cardiac and extracardiac phenotypes than studies conducted on experimental models. Most human variants have a heterozygous dosage, and while experimental models usually examine homozygous variants, data on congenital heart disease obtained from human studies can reveal information about less obvious impacts on heart formation. Furthermore, patients with critical and complex malformations display the greatest frequency of de novo damaging variants in genes linked to CHD, which provides convincing evidence of severe adverse effects on reproductive health and the evolutionary restriction on numerous genes connected with CHD [8,9,10,11,12,13,14,15,16,17,18,19,20,21,22,23,24,25].

**Figure 4:**
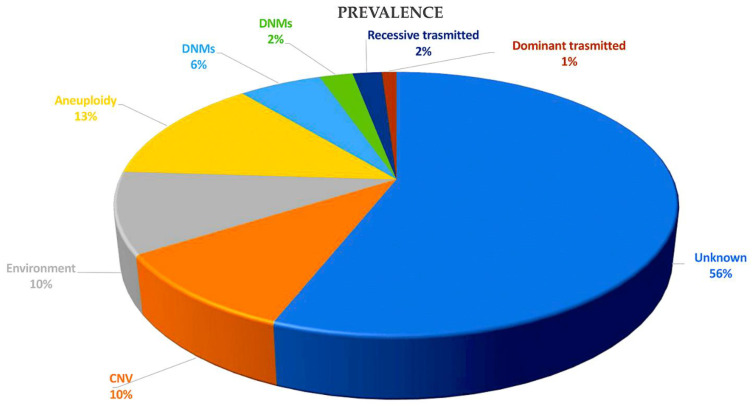
This pie chart illustrates the various causes of CHD and their relative proportions in contributing to the disease. Zaidi et al. [36] were the first to estimate the contribution of de novo mutations to CHD, while Jin et al. [22] presented the first model to systematically investigate inherited CHD mutations. Recessive transmitted causes involve variants in cilia and known CHD genes. Dominant causes of TOF include LOFs associated with *FLT4*, *KDR*, *TBX1*, and *NOTCH1*. DNMs may involve chromatin modifiers (2%) and dominant CHD genes (6%). Aneuploidy aetiology is related to trisomy 13, 18, or 21 and monosomy X. Non-genetic causes of CHD include teratogenic exposures such as alcohol; antiseizure and antiretroviral medication; and illnesses and infectious agents, such as diabetes, hypercholesterolemia, and rubella. The causes of CHD are investigated through gene–gene interactions, gene–environment interactions, polygenic inheritance, and epigenetics. CNVs, including Del22q11.2, Del20p12, Del17q11, Del11q24-25, Del8p23.1, Del1q21.1, Dup12q24, and Dup7q11.23, are also considered. Non-genetic causes of CHD are discussed in References [23,37,38,39,40]. Abbreviations: CHD, congenital heart disease; CNVs, copy number variations; Del, deletion, DMSs, de novo mutations; Dup, duplication; LOF, loss of function.

The question of why CHD is the most prevalent congenital abnormality is intriguing. One hypothesis suggests that the intricate developmental processes involved in heart formation are highly responsive to alterations in gene dosage for many essential genes and pathways. Such changes can cause developmental errors, many of which can be readily detected by the 18-week gestational age screening ultrasonography [26,27]. To showcase the intricate molecular orchestration of heart formation, this review provides a brief summary of cardiac development. Following this, the latest genomic findings and approaches that have been at the forefront of current CHD science over the last decade are discussed. Key signals that govern cardiac morphogenesis have been extensively examined in several informative reviews, which are recommended for further details, including publications [28,29,30,31,32,33,34,35]. Genes with harmful variants found in people with CHD are highlighted in bold during the report (Figure 4, [22,23,36,37,38,39,40]).

## 2. Development of the Heart

The heart develops during the early stages of embryogenesis. The cardiac precursor fields differentiate and coalesce to create the cardiac crescent. During human gestation, the heart tube grows, undergoes regional transformation, and emerges as a nearly fully formed organ by 8 weeks. This process involves a linear and then looped structure. During heart development, differentiating precursor cell clusters interact to generate specialised heart cells with a well-defined three-dimensional architecture within restricted regions.

### 2.1. A Look at the Lines of Progenitor Cells and Their Morphogenesis

**Figure 5:**
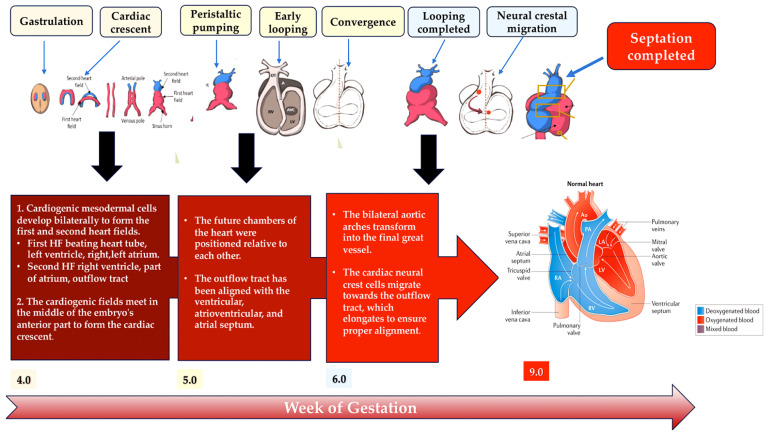
This schematic illustrates the embryonic development of the human heart, including first and second heart field formation, heart tube formation and pumping, looping, neural crest migration, and septation. The process results in a fully developed heart at the end of gestation. Abbreviation: HF, heart field. Refs. [41,42,43,44,45].

The heart is formed by two fields: the first heart field (FHF) from the lateral plate mesoderm and the second heart field (SHF) from the lateral plate splanchnic mesoderm and the migrating cardiac neural crest. These three cell lineages are major contributors to the formation of heart structures in the III, IV, and VI pharyngeal arches [35,41,42,43] (Figure 5). The FHF and SHF, which are symmetrical, merge anteriorly to form the heart crescent, which then coalesces into a linear cardiac tube with ventricular and atrial precursors aligned along the anterior–posterior axis. The heart cells originating from the FHF are involved in the development of the atria and the left ventricle, while the cells from the SHF are involved in the development of the right ventricle and the outflow tract, as well as contributing to both atria. Asymmetric growth results in the ballooning of the external curvature of the cardiac tube. This increases the size of the ventricular chamber [44,45]. The subsequent outflow tract separation and smooth muscle differentiation occur with the contribution of the cardiac neural crest.

### 2.2. Heart Morphogenesis under Genetic Control

Several important biomolecules play a role in differentiating progenitor cells into cardiac cell lineages and directing cardiac morphogenesis. Genes associated with CHD have identified some of these molecules. The FHF arises from the cardiac mesoderm through signalling, including the bone morphogenetic protein (BMP) and fibroblast growth factor (FGF), which are induced by the adjacent endoderm [46,47,48,49]. The expression of key cardiac transcripts required for cardiomyocyte lineage commitment is reduced in experimental models by the deletion of genes encoding BMPs [50]. The expressions of genes such as *GATA4*, *GATA6*, *NKX2-5,* and *TBX5* control sarcomere formation and contraction [28,29,30,31,32,33,51]. Ventricular cardiomyocyte specification is promoted by the expression of *IRX4* in both the FHF and SHF of the cardiac crescent [52]. Factors such as retinoic acid within the mesodermal progenitor fields initiate commitment to an atrial lineage [53,54]. Further specification leads to cardiomyocytes, which populate the left ventricle (*IRX4*, *HAND1,* and *MSX1*) [55,56,57], the right ventricle (*IRX4* and *GATA6*) [55,58], and the outflow tract (*GATA6*, *HAND2*, *ARID3B,* and *TEA* [56,58,59,60]. FGF signalling is required for SHF contributions to the outflow tract, allowing for neural crest migration and the induction of tbx1 expression in the SHF in zebrafish [61]. While there are established differentiation pathways, the progenitor fields in cardiac development still maintain plasticity. For instance, removing FHF cells in zebrafish leads to SHF compensation, which regenerates ventricular cardiomyocytes [62]. Additional pathways and gene networks involved in cardiac neural crest differentiation have been discovered. In mice, the normal differentiation of cardiac neural crest cells is prevented in the absence of retinoic acid signalling [63], which can be ameliorated by maternal vitamin A supplementation [64]. This highlights the genotype–environment interactions in CHD. In contrast, a missense variant of *GATA6*, which is frequently found in patients with CHD and impairs outflow track formation, significantly enhances retinoic acid signalling [58]. *FOXC2* expression is essential for cardiac neural crest migration, as it mediates the activation of Sema3c expression in the SHF. *TBX1* expression, however, results in the counterbalancing of inhibitory signals [65,66].

In the atrioventricular canal and outflow tract, the heart contains non-chambered cardiomyocytes, as well as valvular endothelial and smooth muscle cells. Regulatory proteins, such as *TBX2*, *TBX3* [67], *BMP2*, and *BMP4* [68], repress chamber-specific genes to specify the non-chamber myocardium. In order to populate the endocardial cushions, valvular endothelial cells undergo endothelial-to-mesenchymal transition. This process is controlled by signalling cascades. These include vascular endothelial growth factor (VEGF) [69] and transforming growth factor-β (TGFβ) [70]. Investigations into the differentiation of the outflow tract in mouse models have also shown that histone deacetylase 3 (encoded by Hdac3) is necessary for the differentiation of smooth muscle, whereas FGF and sonic hedgehog signalling are needed for the pulmonary arteries and veins [71,72,73]. Through transdifferentiation, cardiomyocytes can also change into cells that are not cardiac chambers. During the development of the mouse heart, a population of cardiomyocytes was found to transdifferentiate into vascular smooth muscle cells of the great arteries [74].

The left–right signalling axis is established in the embryo within the embryonic node. This occurs through signals mediated by motile and sensory cilia, leading to the asymmetric expression of genes that encode downstream regulatory signals.

Among the genes that encode downstream regulatory signals are those that encode the TGFβ family members Nodal, Lefty1, Lefty2, and Zic3 [75,76,77]. These signals are critical for the correct rightward looping of the linear heart tube, which ensures the correct positioning of the atria, ventricles, and outflow tract and gives rise to the sinoatrial node in the right atrium. Knockout mutations in Lefty2, which encodes left–right axis signalling proteins and transcriptional regulators, or the disruption of Sonic hedgehog or activin signalling in the chick embryo [78] can cause defects in cardiac looping, resulting in congenital heart defects in mice [79].

## 3. Interference with Human Cardiac Development and the Occurrence of Specific Anatomic Congenital Cardiac Disorders

Studying the anatomy of the human embryo has helped to understand how the human heart develops and the causes of cardiac malformations resulting from disrupted spatiotemporal dynamics. CHD is often divided into anatomical subgroups that are the result of abnormalities in common embryonic processes. Therefore, insights into the downstream developmental and physiological consequences will be gained from the discovery of genes with pathogenic variants that cause specific malformations.

- Atrial and ventricular septal defects.

**Figure 6:**
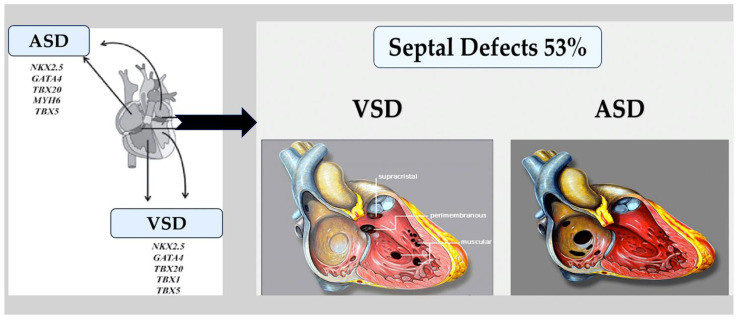
The left diagram shows that two types of congenital heart defects (ASD and VSD) are linked to gene mutations in transcription factors and signalling molecules. The right diagram illustrates abnormal connections between the left and right chambers of the heart, including atrial or ventricular septal defects or atrioventricular canal defects. These defects typically do not result in cyanosis because oxygenated blood moves from the left to the right side of the heart and into the pulmonary circulation. Abbreviations: ASD, atrial septal defect; VSD, ventricular septal defect. The image was created using modified images licensed under a Creative Commons Attribution 3.0 Unported License. Refs. [88,89].

Valve and atrioventricular canal abnormalities, including defects in septum and valve function, may result from the abnormal formation of endocardial cushions and/or dorsal mesenchymal protrusion. Only about 5% of individuals with CHD [80] have complete atrioventricular canal defects. However, almost half of the approximately 40% of individuals with trisomy 21 who have CHD have this defect [81,82]. This association suggests that genes located within a 0.96 Mb region of chromosome 21 play a crucial role in endocardial cushion development [83]. However, the responsible gene(s) is still unknown. As with other syndromes, not all people with trisomy 21 will go on to develop CHD, and up to 60% will have a structurally normal heart [81,82]. The most common congenital heart defects are single ASDs or VSDs; however, the causal aetiology is rarely defined in individuals who have these defects sporadically in the absence of a genetic syndrome. Individuals with rare autosomal dominant familial septal defects were the first to be identified with deleterious variants in NKX2-5 and GATA4. TBX5 variants were identified as the cause of Holt–Oram syndrome in families with septal defects and associated limb defects. In model systems, the proteins of these genes physically interact with each other to promote the correct septation of the heart chambers during the separation of the atria and ventricles [84,85,86,87,88,89] (Figure 6).

- Left-sided obstructive lesions.

**Figure 7:**
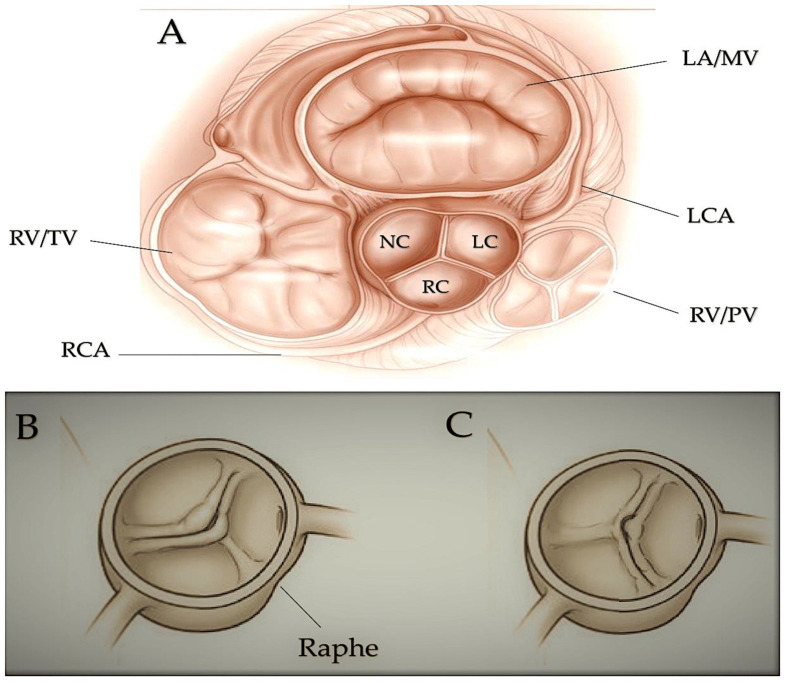
Fused bicuspid aortic valve. Panel (**A**) represents a short-axis normal tricuspid aortic pattern with anatomical proximities. Panels (**B**,**C**) represent two cusp fusion patterns seen in the short heart axis. All BAVs have three sinuses. Raphe structure is between the fused cusps. Non-fused cusp is prominent in respect to the fused ones. (**B**) Left-noncoronary fusion pattern; (**C**) right-noncoronary fusion pattern. The commissure angle of the non-fused cusp has a degree < 180°. Abbreviations: L, left coronary sinus; LA, left atrium; LC, left cusp; LCA, left coronary artery; MV, mitral valve; N, noncoronary sinus; NC, noncoronary cusp; PV, pulmonary valve; R, right coronary sinus; RC, right cusp; RCA, right coronary artery; RV, right ventricle, TV, tricuspid valve. Licenses Centre Cardiologique du Nord (with permission). License Number 5644110549132 License date 8 October 2023; publication NEJM; Title: Mitral valve Repair for Mitral valve prolapse. Ref [91].

**Figure 8:**
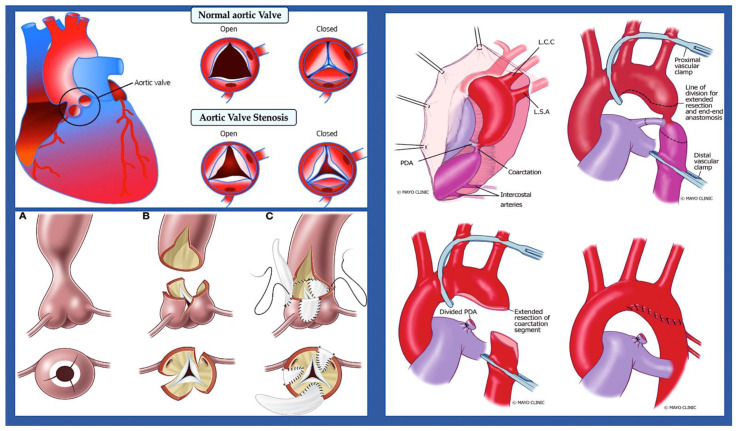
Two examples of left-sided obstructive lesions, represented by a normal and a pathological aortic valve with the corresponding surgical treatment and an aortic coarctation with the corresponding surgical approach. **Top left**: Foetal aortic valve stenosis is a heart condition where the aortic valve, located between the left ventricle and aorta, cannot fully open. This causes blood to flow out of the left ventricle and enlarges the right ventricle, which can lead to poor foetal growth due to changes in blood flow. **Bottom left**: The procedure performed is an extended 3-patch supravalvular aortic stenosis repair. (**A**–**C**) In this procedure, the ascending aorta is transected at its narrowest point, and three incisions are made into the sinuses of Valsalva. (**B**) The sinuses are then enlarged using three pericardial patches, and the patch from the noncoronary sinus is extended into the ascending aorta to ensure symmetrical enlargement of the narrow segment. (**C**) **Top and bottom right**: The diagram illustrates the surgical procedure employed to treat severe aortic coarctation. Extended resection is performed, followed by end-to-end anastomosis. Abbreviations: PDA, patent ductus arteriosus; L.C.C, left common carotid artery; L.S.A, left subclavian artery. The image was created using modified images licensed under a Creative Commons Attribution 3.0 Unported License. Refs. [89,92].

**Figure 9:**
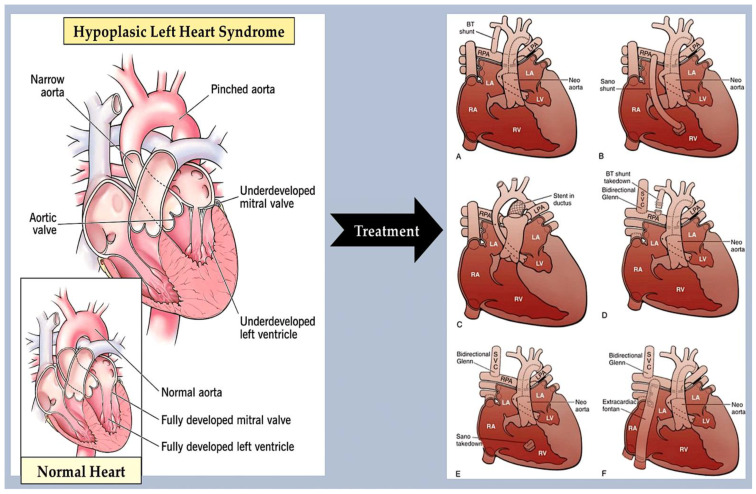
The image shows a comparison between a normal heart (bottom) and a heart (top) affected by hypoplastic left heart syndrome. The different treatment strategies are represented. **On the left:** Diagram of hypoplastic left heart syndrome. The mitral and aortic valves, left ventricular cavity, and aorta are severely underdeveloped, resulting in systemic blood flow being supplied by the patent ductus arteriosus. **On the right**: Staged reconstruction for hypoplastic left heart syndrome. (**A**–**F**) Stage I of the Norwood reconstruction involves using a modified Blalock–Taussig (BT) shunt. (**A**) The second stage is the Norwood reconstruction or the Norwood reconstruction using the Sano modification. (**B**) A hybrid procedure is also an option. (**C**,**D**). The final stage is the Fontan procedure. (**E**,**F**) LA stands for left atrium. LPA represents left pulmonary artery. LV stands for left ventricle. RA denotes right atrium. RPA represents right pulmonary artery. RV stands for right ventricle. SVC denotes superior vena cava. The image was created using modified images licensed under a Creative Commons Attribution 3.0 Unported License. Ref. [89].

This group of CHDs includes several abnormalities that narrow or obstruct the left ventricle and valves. These abnormalities may occur alone, such as mitral stenosis and bicuspid aortic valve (BAV) [90,91] (Figure 7) stenosis and coarctation of the aorta (Figure 8), or in combination [90]. This is a condition known as Shone complex, which is characterised by an annulo-leaflet mitral ring, parachute mitral valve, subaortic stenosis, coarctation of the aorta, and HLHS with mitral and aortic stenosis or atresia and hypoplastic left ventricle (Figure 9) [90]. Compared to other congenital heart defects, left-sided obstructive lesions have a higher heritability. Families with a range of malformations show a higher incidence of CHDs, suggesting a shared genetic pathway in the development of these structures [90,91,92].

BAV (15–30%), aortic coarctation (7–20%), and, less commonly, hypoplastic left heart syndrome [93,94] are common in patients with Turner syndrome (45, XO karyotype). Patients with Turner syndrome have an increased risk of left-sided obstruction lesions due to altered gene dosages on chromosome X and copy number polymorphisms at the 12q13.31 locus [95,96,97]. However, it is currently unknown how these genotypes affect the development of left-sided structures. Jacobsen syndrome is a genetic disorder caused by a deletion in chromosome 11q23 [98]. In 33% of patients, this syndrome can cause severe obstructive lesions on the left side. The development of these lesions is partly due to the requirement of the protein C-ets-1, which is encoded by *ETS1*. This protein is necessary for supporting cardiomyocyte proliferation and extracellular matrix organisation in the developing endocardium [99,100,101]. Bicuspid aortic valve is caused by monogenic damaging variants in *GATA5* [102,103], while hypoplastic left heart syndrome is caused by such variants in *RBFOX2* [104]. Recently, in a family with autosomal dominant BAV, a novel heterozygous GATA6 mutation, p.E386X, was identified [105].

**Figure 10:**
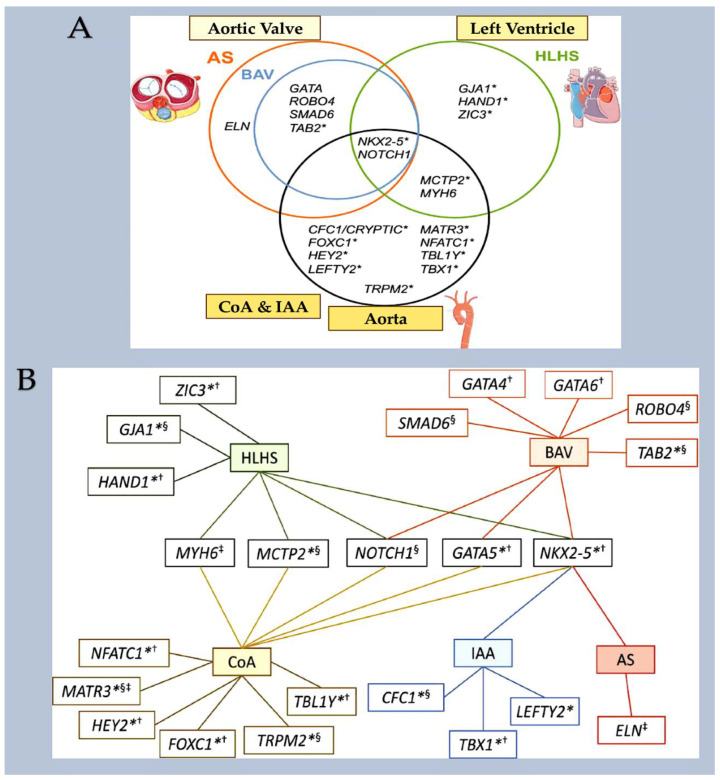
(**A**) A Venn diagram that shows the overlap of genes associated with LSOLs based on disease subtype. Genes without robust evidence of association are marked with an asterisk. (**B**) The genetic landscape of left-sided obstructive lesions. The genes marked with an asterisk have no robust evidence of association. The genes code for transcription factors, structural or contractile proteins, and cell signalling components. Abbreviations: AS, aortic stenosis; BAV, bicuspid aortic valve; CoA, coarctation of the aorta; HLHS, hyperplastic left heart syndrome; IAA, interrupted aortic arch; LSOL, left-sided obstructive lesion. The image was created using modified images licensed under a Creative Commons Attribution 3.0 Unported License. Asterisk denotes genes lacking robust evidence of association. Genes code for ^†^ transcription factors, ^‡^ structural or contractile proteins, ^§^ cell signaling components.

Mutations in *MYH6*, which encodes for the α-myosin heavy chain, can lead to Shone complex [22] and HLHS [106,107]. MYH6 is a protein involved in heart muscle contraction, and it is highly expressed during cardiogenesis. It is one of the few genes associated with congenital heart disease that is not related to transcription or translation (Figure 10).

- Right-sided obstructive lesions.

These conditions result in embryological abnormalities that obstruct the flow of blood from the right side of the heart (RVOT) to the capillary beds of the lungs. Systemic venous blood flow can be obstructed in conditions such as tricuspid atresia, pulmonary atresia with intact ventricular septum, isolated pulmonary valve stenosis, and supravalvular and branch pulmonary artery stenosis. Tricuspid atresia is a condition where the right-sided atrioventricular valve is absent. It is associated with chromosomal trisomy in humans [108] and Zfpm2 LOF in mice [109]. Noonan syndrome, which is caused by variants in the PTPN11 gene [110], is associated with pulmonary valve stenosis in over 50% of children. PTPN11 variants activate mitogen-activated protein kinases (MAPKs) in endocardial cells, resulting in excessive endothelial-to-mesenchymal transition during pulmonary valve development [111,112,113].

**Figure 11:**
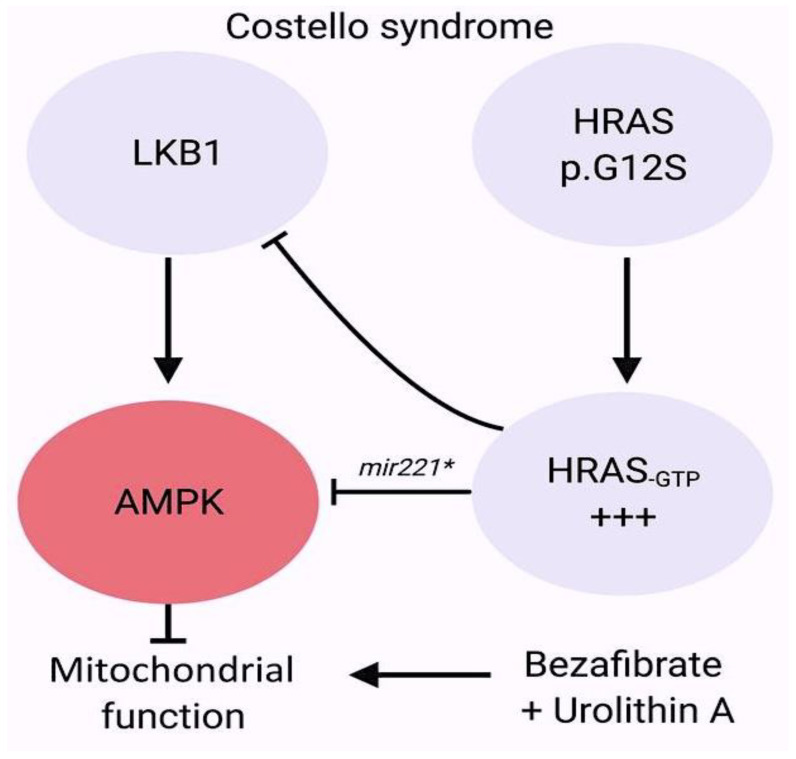
Germline mutations that activate RAS/MAPK signalling cause “RASopathies”. These are a group of rare human developmental disorders affecting over 400,000 individuals in the United States alone. Costello syndrome (CS) is a syndrome of multiple congenital anomalies. It is caused by heterozygous activating germline mutations in HRAS [1]. HRAS hyperactivation inhibits the LKB1/AMPK pathway. Most individuals with CS have a mutation in HRAS at position G12, with over 80% having a p.G12S substitution. Children with CS typically present with increased birth weight, dysmorphic craniofacial features, failure to thrive, and gastroesophageal reflux with oral aversion, especially in the neonatal period. CS can also affect the skin, causing excessive wrinkling and redundancy over the dorsum of the hands and feet, and deep plantar and palmar folds. In addition, individuals with CS have an increased risk of developing benign or malignant tumours. miR-221*, miR-221-5p; +++, increased HRAS-GTP function Ref [115].

The molecular determinants of Costello syndrome (CS) have recently been investigated. Mitochondrial bioenergetics and quality control were activated, resulting in restored organelle function in HRAS p.G12A and p.G12S cell models. Additionally, reduced left ventricular hypertrophy was observed in CS mice, and the incidence of developmental defects was reduced in a CS zebrafish model. These findings highlight the significance of maintaining mitochondrial proteostasis and bioenergetics in the pathophysiology of RASopathies. This suggests that mitochondrial modulators may be beneficial for patients with CS [113,114,115] (Figure 11).

**Figure 12:**
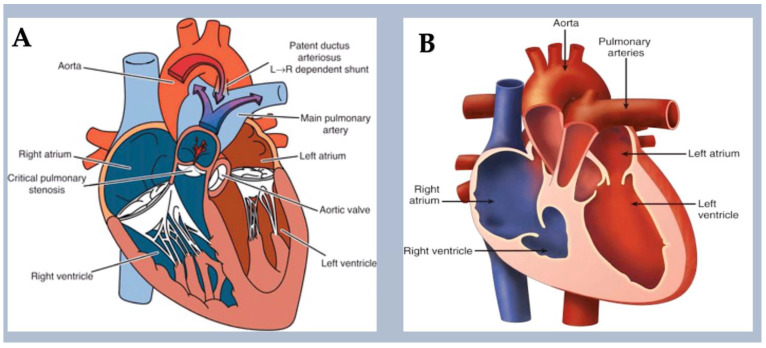
The diagram illustrates a type of obstructive lesion on the right side. (**A**) PA/IVS is an abnormality in which there is complete closure of the communication from the RV to the PA and no ventricular septal defect. (**B**) PA/IVS is a disorder with varying morphology. The RV is often small or hypoplastic, or it can be significantly dilated, as seen in association with Ebstein’s anomaly or TV dysplasia and severe TV regurgitation. Although extracardiac anomalies are unusual in PA/IVS, many different cardiac anomalies can be observed. PA/IVS is typically associated with abnormalities of the right side of the heart, including dysplastic TV leaflets and thickened chordae, which may cause severe regurgitation in utero. Atrial septal defects are also common. It is worth noting that the TV annulus is often small in many cases. In some cases, the tricuspid valve may be fully sealed off, causing tricuspid atresia, or, more commonly, it may be moderately to severely underdeveloped, resulting in tricuspid stenosis. Especially in the setting of a hypertrophied, hypertensive RV with little or no tricuspid regurgitation, coronary abnormalities are common in PA/IVS. An inverse relationship exists between RV size and coronary anomalies—the smaller the RV and tricuspid annulus, the greater the likelihood of coronary anomalies. Abbreviations: PAPA/IVS, pulmonary atresia with intact ventricular septum; PA, pulmonary artery; RV, right ventricle; TV, tricuspid valve. The image was created using modified images licensed under a Creative Commons Attribution 3.0 Unported License. Ref. [89].

Robinow syndrome is a genetic disorder that can cause isolated pulmonary valve stenosis and conotruncal defects, such as tetralogy of Fallot. It can be inherited in an autosomal dominant or recessive manner [116,117,118]. The syndrome is caused by pathogenic variants in genes encoding receptors and ligands controlling planar cell polarity, specifically *WNT5A*, *ROR2*, *DVL1*, and *DVL3* [116,117,118,119]. These findings highlight the significance of planar cell polarity. The pathway of cell polarity affects the development of the cushions in the outflow tract and right ventricular infundibulum in the SHF. Tetralogy of Fallot, PVS, and/or stenosis of the peripheral pulmonary arterial branches can occur in individuals with autosomal dominant Alagille syndrome (caused by mutations in the *JAG1* and *NOTCH2* genes). This suggests the importance of Notch signalling in RVOT development, pulmonary valve development, and vasculogenesis, contributing to the pulmonary arterial tree [120,121,122] (Figure 12).

- Defects in the left–right pattern.

Heterotaxy syndrome is a condition that results in the abnormal positioning of internal organs and congenital heart defects due to anomalies of the left–right axis. This includes structural malformations and aberrant arterial and venous connections. Critical to the patterning of the left–right axis is normal ciliary function. In some patients with primary ciliary dyskinesia, a heterotaxy syndrome with CHD is also present. In patients with nonsyndromic congenital heart disease who have harmful variants in cilia-related genes, transposition of the great arteries is common [123,124]. Patients with cardiac left–right patterning defects also frequently have deleterious variants in genes involved in laterality, such as *NODAL*, *FOXH1*, *ZIC3*, *ACVR2B,* and *SMAD2* [123,124,125]. Defects can cause CHD through autosomal dominant or autosomal recessive mechanisms. Variants in ciliary genes involved in laterality defects can result in CHDs in either autosomal dominant or autosomal recessive inheritance. Variants in genes encoding Nodal and TGFβ signalling proteins are dominantly associated with congenital heart disease, whereas *ZIC3* mutations are associated with hemizygous X-linked heterotaxy syndrome [126,127,128,129,130].

- Conotruncal defects.

**Figure 13:**
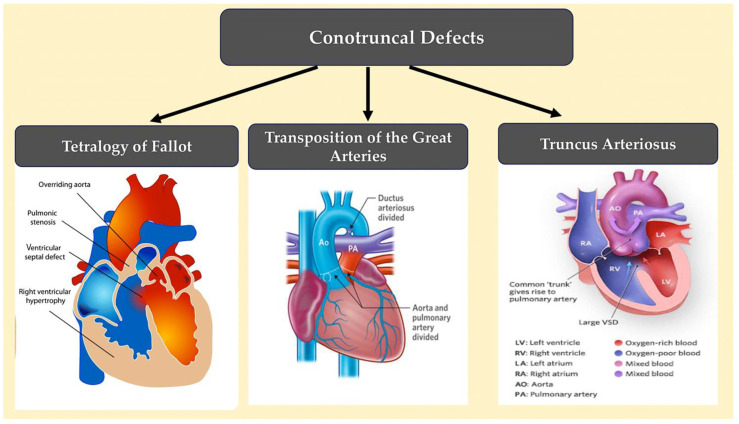
Conotruncal defects include conditions such as tetralogy of Fallot, persistent truncus arteriosus, and transposition of the great arteries, which are caused by the underdevelopment or malposition of the ventricular septum, outflow tract, and/or great arteries. Conotruncal malformations often result in cyanosis due to the shunting of deoxygenated blood into the systemic circulation. The image was created using modified images licensed under a Creative Commons Attribution 3.0 Unported License. Ref. [89].

Conotruncal defects are caused by the underdevelopment or misalignment of the ventricular septum, outflow tract, and/or great arteries, such as tetralogy of Fallot, patent truncus arteriosus, and transposition of the great arteries (see Figure 13).

Autosomal dominant 22q11.2 deletion syndrome often leads to conotruncal defects, which are commonly associated with the deletion of the CHD-associated gene *TBX1* [131,132,133,134]. Similarly, CHARGE syndrome, which is often caused by deletions or variants in the CHD-associated gene *CHD7* [135,136,137,138], can also result in conotruncal defects. These disorders are associated with malformations in the heart, face, and head, which originate in neural crest cells. Harmful gene variants affect cell lineages involved in extracardiac malformations. The congenital heart defects mentioned display variable penetrance and expressivity [138,139,140], indicating that additional factors influence the clinical phenotype. A genetic modifier that influences conotruncal defects in 22q11.2 deletion syndrome has been reported, although these additional factors are largely unknown [131,141]. Repetitive duplications and deletions at 1q21.1, which involve the CHD candidate genes *GJA5* (encoding connexin 40) and CHD1L (linked to the *CHD7* gene), are frequently associated with TOF. Extracardiac abnormalities, such as mental retardation and dysmorphic facial features, may also be present in some cases [142,143,144]. It is important to note that the deletion in the 1q21 proximal region cannot progress towards the thrombocytopenia-absent radius syndrome critical region or the RBM8A gene [144].

Nonsyndromic conotruncal defects are also primarily linked to the monogenic loss of function variants in genes involved in two important signalling pathways that govern the development of processes in the second heart field (SHF) and cardiac neural crest cells. These pathways are Notch signalling (*NOTCH1*) and VEGF signal transduction (*KDR*, *FLT4*, *VEGFA*, *FGD5*, *BCAR1*, *IQGAP1*, *FOXO1,* and *PRDM1*) [145,146,147,148]. The reason why these progenitor fields and developing tissues are particularly susceptible to alterations in the VEGF pathway is still unknown. Conotruncal defects are often caused by autosomal dominant damaging variants in *ZFPM2*, which encodes the transcription factor ZFPM2, as well as in the genes encoding its binding partners, *GATA4* and *GATA6*. These defects are frequently associated with genetic mutations [66,149,150,151,152,153]. The *GATA* genes are transcriptional regulators that act as “pioneer workers” for the transcription process. They bind to and modulate closed regions of chromatin, allowing for the subsequent binding of transcriptional signals. This indicates the importance of the transcriptional regulatory network in developing the conotruncal region [151].

## 4. New CHD Genes Discovered in Cohort Studies

Analyses of CHD cohorts recruited from single centres or through national and international collaborations are increasingly identifying genes associated with congenital heart disease [22,25,154]. The majority of studies include patients with severe or critical CHD. There is an under-representation of patients with prevalent, simple CHD findings and an absence of patients with confirmed genetic syndromes [155]. WES and case–control analyses are commonly used to detect variants. These studies suggest that people with congenital heart disease have similar proportions of total de novo variants to the general population; however, they have significantly higher numbers of rare harmful mutations (allele frequency ≤1 × 10^−5^ in the general population), including a 9% increase in de novo harmful variants, a 7% higher frequency of dominant inherited variants, and a 1% increase in recessive variants [22,23,24,25].

Deleterious mutations are found in an estimated 8–10% of patients with unspecified sporadic CHD [22,25]. The highest rate of de novo harmful variants is found in patients with syndromic congenital heart disease, while the highest rate of heritable LOF variants is found among patients with isolated CHD [25]. The importance of predefined biological pathways in the pathogenesis of CHD has been supported by cohort studies. The newly identified CHD-associated genes include those involved in the Notch [156] and VEGF [156] signalling cascades. However, the most significant functional class of these genes is chromatin remodelling [23,36]. Patients with CHD who have extracardiac anomalies and a neurodevelopmental delay most often have deleterious variants in these genes encoding chromatin remodelling proteins. The broad expression pattern of the genes suggests that organ development uses common epigenetic and transcriptional regulatory pathways.

The importance of ciliary function in cardiogenesis is supported by WES data from patients with CHD [157]. Deleterious mutations in cilia-related genes are often inherited from a healthy parent, as has been shown in other recessive diseases. In contrast, harmful variations in genes associated with chromatin alterations are more likely to occur de novo, which indicates that the damage caused by even a single mutant allele in this class of genes is significant.

- CHD definitive genes.

Studies were conducted on WES data from two cohorts of patients with congenital heart defects: 2056 patients with isolated congenital heart defects, 2994 with syndromic congenital heart defects, and 808 with congenital heart defects and an unknown extracardiac status. The statistical power of the analysis was sufficient to detect a substantial high frequency of harmful mutations in individuals with congenital heart disease compared to their matched counterparts, with correction for analyses of all protein-coding genes (*p* ≤ 2 × 10^−6^) [22,25,158]. In these cohorts, the genes that were identified had a definitive direct link to CHD, as with the genes related to the rare familial forms of CHD. However, a greater contribution to the overall population prevalence of CHD was made by genes identified in large-scale study populations. A total of 18 genes showed significant enrichment in these two WES cohorts, namely, *ADNP*, *ANKRD11*, *CDK13*, *CHD4*, *CHD7*, *DDX3X*, *DYRK1A*, *FLT4*, *KMT2A*, *KMT2D*, *NOTCH1*, *NSD1*, *PACS1*, *PRKD1*, *PTPN11*, *RBFOX2*, *SMAD6,* and *TAB2* [22,25,146,147]. Patients with syndromic congenital heart disease were screened out at enrolment, but mutations in *CHD7* associated with CHARGE syndrome, *KMT2D* associated with Kabuki syndrome, *NSD1* associated with Sotos syndrome, and PTPN11 associated with Noonan syndrome were identified in the study. This suggests that formes frustes of syndromic phenotypes, which escape clinical recognition, may arise from some of the variants associated with syndromic CHD. *CDK13*, *CHD4,* and *PRKD1* are mutated most frequently in syndromic CHDs, whereas *SMAD6* is mutated most frequently in isolated CHDs [159]. Previous studies have emphasised the biological significance of certain genes associated with CHD. In mice, heart malformations have been observed as a result of null mutations in the murine homologues of *SMAD6* and *NOTCH1* [160,161]. *RBFOX2* transcript levels are reduced in some CHD patient tissues compared to in controls [162]. Additionally, the *DYRK1A* protein is a potential therapeutic candidate for treating neurodevelopmental disorders in trisomy 21 patients [163,164]. Recurrent variants in other genes have led to the discovery of new phenotype correlations. In one study, it was found that NOTCH1 and FLT4 mutations are causative factors in TOF, affecting 7% of patients [22,145,146,147]. Additionally, genes previously linked to intellectual disability, such as *ADNP* [165], *ANKRD11* (KBG syndrome) [166], *CDK13* [167,168], and *DDX3X* [169], are now accepted as being implicated in CHD. However, many definitive roles of CHD in heart development remain unknown.

- Genes linked to CHD that have deleterious variants in humans.

WES data suggest that CHD may be influenced by approximately 400 genes, a significantly higher number than currently known [36]. While some of these genes may contain harmful variants in CHD patients, they cannot be classified as definitive CHD-associated genes due to a lack of statistical significance [22,25,146,158]. Morton et al. performed a meta-analysis of whole-exome sequencing data from two large cohorts of patients with congenital heart disease to increase statistical power. The study aimed to improve the understanding of the genetic basis of congenital heart disease. Patients with aneuploidies or deletions at the 22q11 locus were excluded. The study analysed the WES data along with data from a cohort of 128 subjects without congenital heart disease, as well as results from the Genome Aggregation Database. It identified 132 genes associated with CHD, of which 66 had a high frequency of loss-of-function variants and 78 had a high frequency of deleterious missense variants. Patients with aneuploidies or 22q11 deletions were excluded from the analysis [158].

This meta-analysis provides evidence for the involvement of genes associated with coronary heart disease (CHD) in the regulation of transcription. Some of the genes linked to CHD due to loss-of-function variants have functional associations with chromatin remodelling, transcription factors, or RNA processing, and they have been investigated in detail elsewhere. These genes have been implicated in CHD or organogenesis, which explains the associated extracardiac phenotypes. Patients with loss-of-function variants in the group of definitive and candidate genes for the regulation of gene expression in congenital heart disease exhibited a higher incidence of extracardiac phenotypes than those with damaging missense variants [22,25]. The analysis confirmed that new loss-of-function mutations were more common in genes associated with chromatin modification in patients with congenital heart disease and extracardiac abnormalities. Patients with CHD and extracardiac abnormalities showed an enrichment for inherited missense variants. In contrast, patients with isolated CHD showed an enrichment for transmitted LOF variants. These observations suggest that there may be a genetic basis for the presence or absence of extracardiac abnormalities in CHD. It is possible that certain chromatin-modifying proteins have a specific function in the heart.

New technologies have enabled the comparison of results from studies on people of different ethnic and racial backgrounds [22,25,170]. Evaluating harmful missense genetic variations may reclassify some candidate variant genes for CHD as definitive. This will enhance genotype–phenotype correlations and our understanding of their role in heart development.

## 5. Assessing Whole-Genome Sequencing: A New Pathway of Investigation

Modern whole-genome sequencing offers technical advantages over whole-exome sequencing. It eliminates the need for PCR amplification, provides more consistent genome coverage, and has superior validation rates for single-nucleotide polymorphisms and copy number variations [171]. WGS can also identify rare or common mitochondrial sequences, microRNAs, non-coding RNAs, and promoter and regulatory genes, in addition to detecting exomes. WGS has successfully identified *MYH6* variants associated with aortic coarctation in patients. Additionally, adults with tetralogy of Fallot and a missing pulmonary valve have higher rates of deleterious variants in VEGF. A comparison was made between adults with tetralogy of Fallot and a missing pulmonary valve and those with a present pulmonary valve [172,173,174].

### 5.1. Variation in Non-Coding Sequences through Whole-Genome Sequencing

Only 1% of the genome codes for proteins, with the majority (99%) being non-coding sequences. These include promoters, enhancers, and factors that influence chromatin topology or the accessibility of RNA or protein factor binding sites. Other unknown factors may also be involved [175,176,177,178,179,180]. Non-coding sequence variants can be harmful and may be the source or modifier of the expression of genes associated with CHD. Topological association underlies this hypothesis. During cardiomyocyte differentiation in vitro, there is evidence of a relationship between non-coding regions and gene promoters [181,182,183]. Additionally, assessing non-coding sequences within CNVs can aid in defining them. The regions involved in CHD are likely to be those that are most involved in the development of CHD [184]. In fact, targeted analyses of non-coding sequences in *TBX5* flanking regions were analysed, and three potential enhancers were identified. One of these enhancers contained a homozygous variant in a patient with CHD [185]. The homozygous non-coding variant prevented cardiac expression in model organisms when compared to the normal enhancer sequence [185].

A study analysed de novo variants in non-coding sequences in congenital heart disease, with a focus on those likely to regulate gene expression. Two related techniques were used [186,187]. Variants were detected by the sequencing of whole genomes based on scoring obtained from a machine learning evaluation of various genomic features or by their position within regions of transcription activity during the differentiation of human induced pluripotent stem cells (hiPSCs) into cardiomyocytes (hiPSC-CMs).

Functional assays were used to validate the non-coding variants prioritised by both strategies. Further analysis showed that, in patients with CHD, compared to controls, there was an increase in non-coding variants within the binding sites of RNA-binding proteins. These results point to a potential impact. Non-coding variants, in particular those found in promoters and at RNA-binding protein sites, may account for between 4 and 12 per cent of CHD cases. However, to confirm and extend these initial findings, further analysis of larger cohorts is needed [186,187].

### 5.2. Using Whole-Genome Sequencing to Identify Structural Variation

WGS allows for the detection of structural mutations with a higher resolution than existing methods and increases the repertoire of LOF mutations identified in human disease [188,189,190]. Earlier work has suggested that small structural mutations can lead to CHD. These include an 8 kb deletion in *FLT4* linked to tetralogy of Fallot [147] and a 10 kb deletion in *CHD7* associated with CHARGE syndrome caused by the insertion of an Alu element. Genomic inversions and complex rearrangements can be identified by WGS [18,19,20,21,22,23,24,25]. These structural mutations have been reported in patients with unexplained Alagille syndrome, affecting the expression of the *JAG1* and *NOTCH2* genes. It is expected that the number of deletions, insertions, translocations, and other genomic rearrangements identified in CHD will increase significantly with advances in structural variant algorithms for analysing whole-genome sequencing [145,146,147,191,192].

### 5.3. Future Uses of Genomics

Undiscovered genomic features offer exciting opportunities to improve our understanding of the genetics of CHD. They may explain the 55% of cases of congenital heart disease that remain unexplained. Whole-genome sequencing facilitates the investigation of the oligogenic causes of CHD, including both uncommon and common variants, by providing a more complete genotyping of an individual’s SNPs and structural variants. Extended testing of genetic variants across the genome may provide insights into the penetrance and phenotypic diversity of congenital heart disease, both in cases of familial inheritance and in unrelated patients with a common CHD genotype. Additional research is required to comprehend the impact of environmental exposures on the consequences of a CHD genotype. This is due to the significance of the following factors: CHD-associated genes and chromatin remodelling have a strong correlation, and environmental factors can also affect chromatin structure [193,194]. Some puzzling features of CHD may be elucidated by exploring these complexities. For instance, epidemiological data suggest that familial recurrence rates of CHD are between 3% and 7%, lower than those predicted based on current evidence on CHD genotypes. Recurrence rates of 25% and 50% would be expected for autosomal recessive and dominant conditions, respectively [195,196,197].

## 6. Deriving Causality by Integrating Biology

In the clinical setting, identifying genes and variants with a potential causal relationship with congenital heart disease, along with investigating gene regulation during development and conducting functional investigations in model systems, is crucial for establishing causality.

### 6.1. Insights Gained from Single-Cell Transcriptomics

A comprehensive analysis of the many cells present in both the developing mouse heart [198] and the mature human heart [199,200] has become possible with the advent of single-nucleus RNA sequencing and single-cell RNA sequencing (scRNA-seq).

These studies have revealed differences in gene expression between various cell lineages and unique cell states within each lineage that were previously unknown when analysing bulk RNA sequencing data. For instance, the healthy human heart consists of four ventricular and five atrial cardiomyocyte subsets in healthy adults. These findings have raised new questions, and ongoing studies are investigating the developmental origins, plasticity, and physiological functions of these different cell subsets. This evidence provides valuable insights into the cellular composition of the heart during foetal development. Transcriptional signatures of cardiomyocytes (*TNNI3* and *TNNT2*), fibroblast-like cells (*COL1A1*, *COL1A2,* and *POSTN*), endothelial cells (*PECAM1* and *KDR*), and valve cells (*SOX9*) have been detected in recent scRNA-seq investigations of human foetal hearts [201]. During gestational week 7, genes related to the Notch signalling pathway are significantly more abundant in endocardial cells, which coincides with the compaction of the myocardium. Meanwhile, genes related to the BMP signalling pathway are expressed in endocardial and fibroblast-like cells from the 5th to the 25th weeks of gestation, indicating periods of endocardial-to-mesenchymal transition [201].

A groundbreaking paper has published an analysis of scRNA-seq data from the developing mouse embryo, adding to the limited data available from human tissues. It describes the complexity and transcriptional profiles of lineages derived from three germline layers: the early lateral plate mesoderm, paraxial mesoderm, and neuronal mesoderm [198]. From embryonic day (E) 6.75 to E7.5, *Mesp1* expression in mouse cells marks the disappearance of pluripotency and the acquisition of a commitment to the cardiac lineage [202]. Initially, *Hand2* expression specifies the second heart field, but, later, it also specifies cells of the LVOT and RVOT. At E9.5 and later stages, cardiomyocytes stop dividing and begin to exhibit gene expression patterns that are specific to each chamber. The datasets reveal significant crosstalk between cell lineages that drive cardiac differentiation and morphogenesis. These resources provide more detailed information on the temporal and lineage-specific expression of genes containing harmful variants linked to CHD than is possible from transcriptional analyses of bulk cardiac tissues [203]. Pijuan-Sala et al. reported significant findings on the presentation of single-cell RNA sequencing data from embryonic germ layer derivatives that contribute to heart development in mice [204]. These genes reveal the temporal emergence of cardiomyocytes, endothelial cells, and neural crest cells. They highlight the role of definitive and candidate CHD genes previously discovered in the meta-analysis. They reveal diverse lineage-specific and temporal expression in embryonic cells that are critical for normal cardiovascular development. As anticipated, genes with harmful variants found in patients with tetralogy of Fallot-associated CHD, such as *Kdr*, *Flt1*, *and Flt4*, exhibit a high expression in E8.25–E8.5 endothelial cells, in line with the known functions of the encoded proteins in the VEGF pathway [145,147]. In contrast, three genes, *Rpl19*, *Rps24,* and *Rps26*, which have been implicated in Diamond–Blackfan anaemia and CHD, have different cellular and temporal patterns in the developing mouse heart. At E7.5-E7.75, *Rpl19* and *Rps26* are enriched in the mesenchyme, while *Rps24* is expressed in cardiomyocytes at E8.5 [205]. It is possible that these genes play different roles in cardiac development due to their enrichment in cardiomyocytes at E8.5. Finally, genes that encode chromatin-modifying proteins, which play an important role in heart development and have been found to be affected by harmful mutations in CHD patients (*Chd4*, *Chd7*, *Kat6a*, *Kat6b*, *Kmt2*, and *Kmt2d*), have varying cellular and temporal expression profiles during mouse heart development [182]. Altered transcriptional dynamics in many cell types are likely to contribute to CHD. Datasets will help to understand the role of the poorly studied genes enriched in diseased patients and identify disrupted pathways, according to Morton et al. [158,187]. These insights will enhance our knowledge of the pathological effects of deleterious variants and aid in the elucidation of CHD. In patients with CHD, cellular deficiencies may contribute to lifelong adverse events [182,183,206].

### 6.2. Findings from Pluripotent Stem Cell Models of Congenital Heart Disease

Individual or isogenic lineages can be used to derive HipSCs. CRISPR-Cas9 is a tool for the correction of harmful variants in patients with congenital heart disease. The process of distinguishing hiPSC-CMs shares similarities with the FHF [207,208] or SHF, cardiac neural crest, or pre-valvular endocardial cells [209,210,211]. Combined with epigenetic and transcriptional profiling, investigators can analyse early developmental stages to assess the differentiation of mesoderm progenitor cells into early cardiomyocytes [178]. For instance, this technique can be used to study the differentiation of mesoderm progenitors into hiPSC-CMs, which is challenging to achieve in model organisms [58,209,210,211].

Molecular characterisation can be performed on mutant hiPSCs to assess the functional impact of CHD-associated variants on gene expression, chromatin status, and differentiation states. hiPSC-CMs have provided valuable insights into the pathogenesis of CHD, although these in vitro assays do not take into account the effects of in vivo morphology and haemodynamics. Disruption of key downstream regulators associated with outflow tract development, specifically *KDR* and *HAND2*, is present in patients with GATA6 LOF variants. This disruption explains why GATA6 LOF variants are associated with outflow tract malformations in a study using hiPSCs [58]. In contrast, hiPSCs carrying a recurrent GATA6 missense variant found in patients with CHD and pancreatic agenesis exhibit a significantly altered epigenetic landscape and increased retinoic acid signalling [58]. Transcriptomic analyses of *TBX5*-haploinsufficient hiPSCs revealed that gene regulatory networks controlling cardiomyocyte differentiation are sensitive to *TBX5* dosage. Additionally, novel genetic tools that may affect CHD phenotypes have been identified [212]. The iPSC-CMs of HipSCs with a missense variant in *GATA4*, which is linked to septal defects and pulmonary stenosis, display a lack of *GATA4-TBX5* interactions and an incorrect expression of endothelial cell genes [84].

The potential of human induced pluripotent stem cells in assessing non-coding variants in congenital heart disease is significant. This approach overcomes the challenge of the limited conservation of non-coding sequences between species, which hinders analysis in experimental models. Large-scale parallel assays developed for hiPSCs can distinguish likely contributing non-coding variants from numerous benign polymorphisms, helping to identify the associated genes [213]. Profiling the epigenetics and transcription of hiPSCs during cellular differentiation can provide valuable insights. Achieving early cardiogenesis in model organisms is a challenging task [182].

## 7. Moving Genomics into the Clinical Setting

Due to advances in testing methods and the identification of associations between genotypes and clinical phenotypes over time, the field of the clinical genetic screening of people with CHD is constantly evolving. Genetic testing for congenital heart disease has traditionally targeted syndromic CHD or selected CHD phenotypes that are closely linked to a particular genotype (such as the 22q11 deletion) [214]. However, clinical whole-exome sequencing is becoming increasingly available and cost-effective, allowing for the gene-based diagnosis of both syndromic and nonsyndromic congenital heart disease. As WES provides a comprehensive analysis to identify the causes of CHD, as well as incidental findings that are clinically actionable and/or of unclear relevance, informed consent and guidance from genetic counsellors or experienced clinicians is essential. In addition, as new information arises, it is important to establish criteria for determining the pathogenicity of CHD. These criteria should be similar to those proposed by ClinGen and the American College of Medical Genetics and Genomics [215,216,217,218].

Patient care can be significantly impacted by the identification of definitive causal variants for congenital heart disease. Genotyping can inform the risk of recurrence, prenatal genetic counselling, and pre-implantation genotyping. This can benefit both families of newborns with CHD and adults with repaired or mitigated CHD [219]. The genotype can increasingly indicate the risk of adverse extracardiac conditions or comorbidities, such as neurodevelopmental delay [220,221]. This emphasises the importance of monitoring negative postoperative results [220,221] and the potential development of cancers [222,223,224].

### 7.1. CHD in Association with Extracardiac Abnormalities or Neurodevelopmental Disabilities

The probability of identifying a pathogenic CHD genotype rises from 8% to 22% when an extracardiac anomaly or neurodevelopmental delay is present, particularly in genes that modify chromatin [22]. In cohorts with neurocognitive disorders, harmful variants are more likely to be found in chromatin-modifying genes [23,225,226,227], indicating a significant genetic contribution to the association. It is important to note the specific genetic factors that contribute to this association between CHD and neurodevelopmental delay. Identifying chromatin-related damaging genotypes in newborns with CHD can inform pre-emptive interventions to improve their learning and social skills [225,226,227].

### 7.2. De Novo Copy Number Variations Have an Impact on Perioperative Outcomes

Congenital heart disease genotypes can aid in identifying high-risk patients for heart surgery. Patients with CHD and 22q11.2 deletions experience longer durations of cardiopulmonary bypass and more reoperations. Additionally, CHD patients with other large CNVs (>300 kb) have a higher risk of death or transplantation and longer stays in intensive care than those without the deletion [228,229,230,231,232]. WES is continuing to extend these findings. Patients with congenital heart disease who have new genetic mutations or harmful genetic changes experience longer time on a breathing machine and reduced survival without a heart transplant [220]. These negative outcomes are particularly significant for CHD patients without extracardiac anomalies. Some genomic regions associated with risk are linked to cardiac trabeculation and myocardial performance, which may underlie the risk of heart failure and transplantation in some patients with CHD [233]. The link between genotype and morphological structure is important to consider.

### 7.3. Cancer Risk in Patients with Congenital Heart Disease

The incidence of cancer is 1.4 to 2 times higher in adults with CAD than in the general population [222,223,224]. Certain CHD genotypes may also increase the risk of cancer [152] in combination with the increased radiation exposure associated with therapeutic interventions [234,235]. Many genes associated with congenital heart disease also have harmful variants associated with cancer risk. Patients with CHD who have harmful variants in these genes often have extracardiac manifestations. This association may be related to the widespread developmental role of some CHD-associated genes.

### 7.4. Forthcoming Applications in the Clinic

Investigating the relationship between the causal variants associated with CHD and clinical outcomes presents a significant opportunity. This text explores how genetic variants or defects in signalling pathways contribute to the variability in CHD phenotypes. It also raises questions about the impact of genetic variants associated with CHD on survival rates after surgical procedures and the relationship between variants causing structural heart disease and long-term cardiovascular function measures in CHD patients. To answer these questions, large genome-wide sequencing datasets are required. Collaborative efforts between multiple centres are likely to be essential to harmonise these datasets with comprehensive, high-quality clinical data. This is due to the cost and size of the datasets required to answer these questions.

## 8. Conclusions

The genetic architecture of CHD has been discovered through technological advances. This covers harmful genetic variations in both the germline and mosaic states, as well as the copy number and structural variations that impact multiple genes, and variations in non-coding sequences. These findings have implications that extend beyond the comprehension of the molecular basis of this prevalent congenital anomaly. These observations have identified significant genes and pathways that are involved in the formation and operation of the heart and other organs. Genetic variants associated with CHD also increase the risk of neurocognitive dysfunction and cancer, and they confer specific risks to patients requiring surgical intervention for CHD. Although progress has been made in understanding the genetic architecture of CHD, there is still much to learn. The causes of CHD remain unknown in over 50% of patients. To address this challenge, we need to explore new directions. Identifying abnormal gene expression is a potential avenue to increase productivity. Direct analyses of human CHD tissues, comprehensive assessments of non-coding sequences and oligogenic variants, and examinations of the effects of environmental exposures may be useful. The results of these studies offer promising improvements not only for the diagnosis, classification, and clinical care of patients with CHD but also for enriching our understanding of developmental and genomic biology.

The understanding of the genetic causes of cardiomyopathies has contributed enormously to the knowledge of the molecular basis and pathophysiology of hypertrophic, dilated, arrhythmogenic, restrictive, and left ventricular non-compaction cardiomyopathies. The diversity of the pathways contributing to pathological cardiac remodelling has been demonstrated by the identification of more than a thousand mutations in approximately 100 genes encoding proteins involved in different subcellular systems. Furthermore, the traditional perspective, which was based on morphology and physiology, has now been replaced by genetic and molecular patterns that define the causes of cardiomyopathies. Currently, advanced genetic technologies offer the possibility of diagnosing individuals based on their genetic findings, sometimes even before the clinical symptoms of the disease manifest. However, translating the rapid pace of research and the complexity of genetic information into clinical practice requires expert counselling and analyses of genetic test results to manage families with inherited cardiomyopathies [236].

## Data Availability

Not applicable.

## Abbreviations

A, atrium; Ao, aorta; ASD, atrial septal defect; AV, atrioventricular; BAV, bicuspid aortic valve; CoA, coarctation of the aorta; DA, ductus arteriosus; DORV, double-outlet right ventricle; HLHS, hypoplastic left heart syndrome; LA, left atrium; LCA, left carotid artery; LSCA, left subclavian artery; LV, left ventricle; PA, pulmonary artery; PTA, persistent truncus arteriosus; RA, right atrium; RCA, right carotid artery; RSCA, right subclavian artery; RV, right ventricle; TGA, transposition of the great arteries; TOF, tetralogy of Fallot; V, ventricle; VSD, ventricular septal defect. From Ibrahim et al. *Int. J. Mol. Sci.* **2023**, *24*(22), 16258; https//doi.org/10.3390/ijms242216258 [1].
